# Development of Robust PEBAX-Based Angiographic Catheter: Design and In Vitro Study

**DOI:** 10.3390/ma17174248

**Published:** 2024-08-28

**Authors:** Hafsa Inam, Murtaza Najabat Ali, Ibraheem Raza Jameel, Dil Awaiz, Zunaira Qureshi

**Affiliations:** 1 Biomedical Engineering and Sciences Department, School of Mechanical and Manufacturing Engineering (SMME), National University of Sciences and Technology (NUST), Islamabad 44000, Pakistan; hafsainam91@gmail.com; 2 N-ovative Health Technologies, NHT, National University of Sciences and Technology (NUST), Islamabad 44000, Pakistan; ibrahimraza.ps@nhtpl.pk (I.R.J.); dilawaiztto@nhtpl.pk (D.A.); 3 Medical Devices Development Center (MDDC), National University of Sciences and Technology (NUST), Islamabad 44000, Pakistan; amr@mddc.nust.edu.pk

**Keywords:** PEBAX^®^, angiographic catheter, angiography, braided catheter, corrosion resistant, burst test, leak test, tensile strength, flow rate, performance testing

## Abstract

Background: Keeping in mind the unceasingly escalating prevalence of coronary disease worldwide, the mortality rate is also expected to rise with a staggering increase in healthcare costs. Angiography is the gold standard for diagnosing these blockages that trigger these diseases. Amides and urethanes are the common catheter construction material used for angiography. However, the experimental evidence verifying the use of PEBAX^®^ and comparing its performance with that of commercially available catheters for angiography is not published despite it being well recognized for its excellent flexural modulus, mechanical properties, and biocompatibility and its potential to reduce the incidence of vascular spasm during intravascular diagnostic and interventional procedures. Therefore, the aim of this study was to develop a PEBAX^®^-based angiographic catheter and evaluate its performance in comparison with three commercially available nylon- and polyurethane-based angiographic catheters. Methodology: A PEBAX^®^-based angiographic catheter was developed for this purpose. This study analyzes and reports the performance and behavior of PEBAX^®^-, nylon-, and polyurethane-based catheters. The catheter’s performance and arterial forces’ endurance nature were mapped out by evaluating pushability (advancement force) and selective bench tests outlined in the applicable regulatory standard. Conclusions: The PEBAX^®^-based catheter exhibited the least bond-flexural rigidity (180.4 g), which was approximately one-third of that shown by all six French catheters and which exhibited the least advancement force (510.4 g), which was approximately 50% less than that of the nylon- and polyurethane-based catheters when traversing through the mock arterial system. Bench testing was carried out as per the applicable regulatory standard; the differences obtained between individual catheters were discussed in detail. Based on this extensive in vitro assessment, it was concluded that the PEBAX^®^-based catheters outperformed the nylon- and polyurethane-based catheters, exhibiting an exceptionally minimal advancement force of 510.4 g. This leads to the inference that this catheter can inject more radiopaque material (because of the enhanced flow rate) to the coronary arteries and can play a significant role in minimizing vascular spasms during a diagnostic procedure.

## 1. Introduction

Heart disease is the leading cause of death worldwide. According to the World Health Organization, ischemic heart disease claimed the lives of roughly 17.9 million people in 2019 [1]. The mortality trend attributable to coronary artery disease has been contrary in emerging and developed nations [2,3]. Atherosclerosis is plaque formation on the inside walls of arteries caused by cholesterol and fatty depositions, which obstruct the blood flow to the heart [4,5,6,7]. Coronary angiography is a procedure that employs fluoroscopy to examine the blood channels of the heart. This procedure helps determine whether the coronary arteries are obstructed or narrowed because of plaque [8,9,10]. During this procedure, a flexible tube, commonly known as a catheter, is injected into the femoral or radial artery. The radiopaque tip of the catheter serves as a guiding aid. An angiographic catheter injects a radiopaque dye to acquire angiographic scans by illuminating the blood arteries when the radiopaque medium flows through them [11].

In 1947, Radner was the first person who performed coronary angiography radially (through the radial artery) by using a cut-down procedure [12]. In 1989, Lucien Campeau discovered the radial approach for coronary interventional procedure, leading to 100 cases concerning the radial angiographic technique [13]. Kiemeneij supported the whole concept and practice [12,14]. Compared to the femoral route, radial access (through the radial artery) for coronary angiography and angioplasty has fewer problems (such as vascular spasms). The radial method also reduces unfavorable clinical effects, such as hemorrhage after clinical interventional procedures [15,16]. As a result, radial coronary angiography has become the gold standard for diagnosis before performing a percutaneous coronary intervention [17].

Medical devices used in diagnostic and interventional practices have evolved over decades because of the progress and innovation in biomaterials and medical device design [18]. A good understanding of catheter design and engineering is needed to produce optimal-quality catheters. Cardiologists are enthusiastic about the reduced frictional properties of intravascular catheters inserted into blood vessels. The insertion of diagnostic angiographic catheters into the human body is not only painful but can also cause blood vessel disruption if the devices have a high friction coefficient [18,19]. Furthermore, flexibility and pressure endurance are critical while supplying radiopaque material to the coronary artery. Flexible tubing, kink resistance to withstand artery compressional stresses, and pushability are all functional needs in a catheter design. Polymers such as polyurethane (PU), nylon, the polyamide family, PEBAX^®^, and PTFE (polytetrafluoroethylene) are used as outer and inner layers in braided, reinforced catheters [13].

PEBAX^®^ is a low-density, low-weight thermoplastic and its customizable nature outperforms practically all other thermoplastic elastomers, allowing features such as hardness, chemical resistance, and even processing to be fine-tuned [13,14,15,20]. The PEBAX^®^ family’s combination of essential qualities, including high pushability, flexibility, and tailorability, makes it suitable for catheter building applications [14]. PEBAX^®^ is usually acknowledged as a urethral catheter material of choice because of its inert nature and ability to manufacture multi-durometer lengths [15]. It also has a slightly greater flexural modulus than many TPEs; therefore, it outperforms them in kink resistance tests. Since a catheter may require varied hardness along its length, small sections of a hard PEBAX^®^ grade followed by a soft PEBAX^®^ grade can be used [15].

PEBAX^®^ was carefully selected as the braided tube polymer layer for constructing an angiographic catheter because it appears to be an appealing choice due to its biocompatible properties (low cytotoxicity) and its customizable physicochemical properties. The properties of PEBAX^®^ may be tailored to construct catheters of varied flexibility, ranging from very hard and unyielding to a very soft flexible polymer, by rearranging polymeric chain blocks and their corresponding ratios [19]. These unique polymeric qualities preserve the right combination of toughness traditionally associated with polyamides and of flexibility and elasticity associated with polyether and polyesters [16,17,21].

This study is intended to develop and evaluate the performance of a PEBAX^®^-based angiographic catheter in comparison with three commercially available nylon- and polyurethane-based angiographic catheters. This research concentrates on the in vitro testing and outcomes of PEBAX^®^-based diagnostic angiographic catheters rather than the novelty of PEBAX^®^ as a material for catheter assembly. A PEBAX^®^-based angiographic catheter was designed and subjected to physical performance testing, comparing it with standard catheters. The evaluation followed the guidelines outlined in ISO-10555-1:2013 [18] and included assessments such as corrosion resistance, flexural strength, as well as the trackability and pushability of the catheters.

## 2. Materials and Methods

### 2.1. Materials

PEBAX^®^ (sourced from CUUMED Catheter Co, Taipei, Taiwan) was used to design and manufacture extruded polymeric tubes (proximal and distal end of the catheter), and then configured into an angiographic catheter to diagnose plaque deposition in coronary arteries. The braiding and extrusion processes were used to produce PEBAX-based diagnostic angiographic catheter samples. PEBAX^®^-based catheters with distal tip shapes were also prepared in the lab for mechanical testing and analysis. All the protocols were developed per the ISO 10555-1:2013/Amd 1:2017 international standard, as suggested by FDA (Food and Drug Administration) guidelines [18].

Cordis’ Infiniti^®^, BBraun’s Angiodyn^®^, and Terumo’s Radifocus Optitorque^®^ were obtained from commercial sources to be used as standard materials.

### 2.2. Development of PEBAX^®^-Based Diagnostic Angiographic Catheter and Description of Prototype

**Figure 1:**
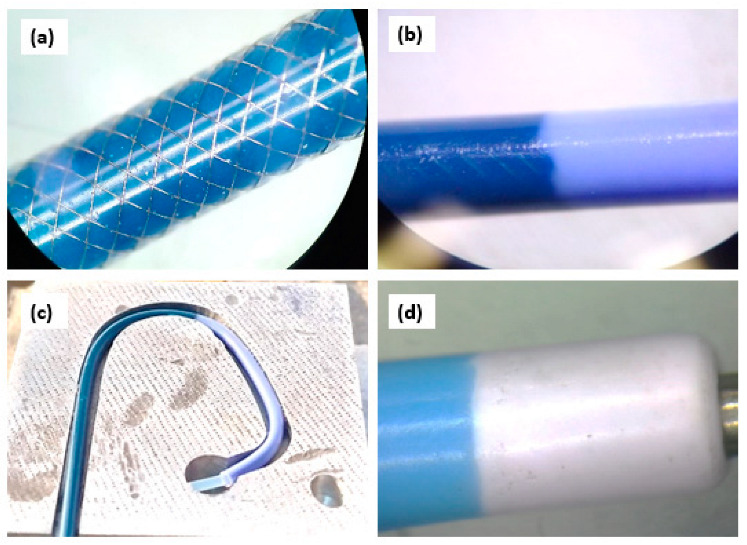
(**a**) Braiding and braiding pattern over PEBAX^®^, 7233, 72D inner liner. (**b**) Welding of braiding to non-braiding part. (**c**) Distal shaping of 6F PEBAX^®^-based catheter, Judkins Left, placed in a die before thermal exposure. (**d**) The tapered tip end of the catheter.

A 16-wire braiding unit was used to braid the catheter reinforced shaft, also known as the braided shaft/sandwich layer. A single, round wire, made up of stainless steel 304, having a diameter of 53 ± 0.01 um, was used for braiding. A total of 55 picks per inch were kept constant throughout the braiding of the reinforced shaft; 16 wires were involved in the braiding of the reinforced catheter, making a pattern of two-over-one throughout the braided shaft, as shown in Figure 1a. The braided shaft’s outer jacket was of the same specs as those of the inner liner, which was extruded in a continuous process. The inner layer of the braided shaft was made of PEBAX^®^ 7233, having a shore hardness of 72D, and was extruded at 235 °C ± 10 °C using CUUMED medical tube extruders (Taipei, Taiwan). The non-braided tube at the distal end was made of PEBAX^®^ 5233 by extrusion at 220 °C ± 10 °C, having a shore hardness of 55D. The soft tip was made of PEBAX^®^ 3533 by extrusion at 205 °C ± 10 °C, which had a shore hardness of 35D. The outer diameter of the whole shaft was 2.00 ± 0.05 mm and the inner diameter was 1.40 ± 0.05 mm, which equated to 6 French of a diagnostic catheter. All the extruded tubings were subjected to cooling in water right after extrusion. After cooling, the tubings were cut into the required lengths and were then subjected to welding to construct a single unit of a PEBAX-based diagnostic angiographic catheter. Briefly, a braided tube with a length of 900.0 ± 0.5 mm was overlap welded to a non-braided tube having 100.0 ± 0.5 mm length (Figure 1b) and a 3 mm soft tip fused and tapered at the distal end (Figure 1d). A thermoforming machine was used to shape the catheter’s distal end. The catheter was positioned in aluminum die, designed for a Judkins Left (Figure 1c). The die was heated to 120 °C for 120 s and cooled for 200 s. The shaped catheter was eventually withdrawn from the die.

#### Description of Tests Related to ISO 10555-1:2013/Amd 1:2017

The ISO 10555-1: 2013/AMD 1:2017 standard states the general requirements for intravascular catheters delivered in sterile packaging and meant for single use only. Tests were conducted in compliance with Annex A, B, C, E, and F of ISO 10555-1:2013/AMD 1:2017.

### 2.3. Test for Corrosion Resistance

This test was performed to observe the corrosion resistance ability of PEBAX^®^-based and commercial catheters. The purpose of this test was to evaluate if the metallic elements embedded in the catheter, whose intended use is to supply a media delivery path, exhibited any sign of corrosion when immersed in a salt solution. The execution of the test in the laboratory involved the use of borosilicate beakers filled with saline solution (1 L in each beaker) and maintained at 25 °C. The diagnostic catheter (100 cm length, 1.67 mm OD (5 Fr), and 2.00 mm OD (6 Fr) was immersed in the different saline solutions for 5 h. After immersion, the PEBAX^®^-based catheters were removed from the beaker. The beakers were then filled with distilled water at a boiling temperature. The diagnostic catheter was again immersed in water for 30 min. Following this step, water was allowed to cool to 37 °C. The catheters were kept in water at 37 °C for 48 h (about 2 days); after removal from the water, they were allowed to dry at room temperature. The sample was inspected for corrosion using a Leica Optical Microscope with a 25 × Lens (13 microns per division, readability).

#### 2.3.1. Liquid Leakage under Pressure

This test was performed to ensure that the luer lock or any welded section of the catheter did not leak when subjected to pressure. The catheter hub was connected to a leak-proof connector and was maintained at 300 kPa for 30 s during testing. The diagnostic catheter shaft was fixed on the table, and the welded areas were submerged into water to observe any leakage. The PEBAX^®^-based catheters along with BBraun’s Angiodyn^®^, Cordis’ Infiniti^®^, and Terumo’s Radifocus Optitorque^®^ were also subjected to the same pressure.

#### 2.3.2. Determination of the Flow Rate through the Catheter

**Figure 2:**
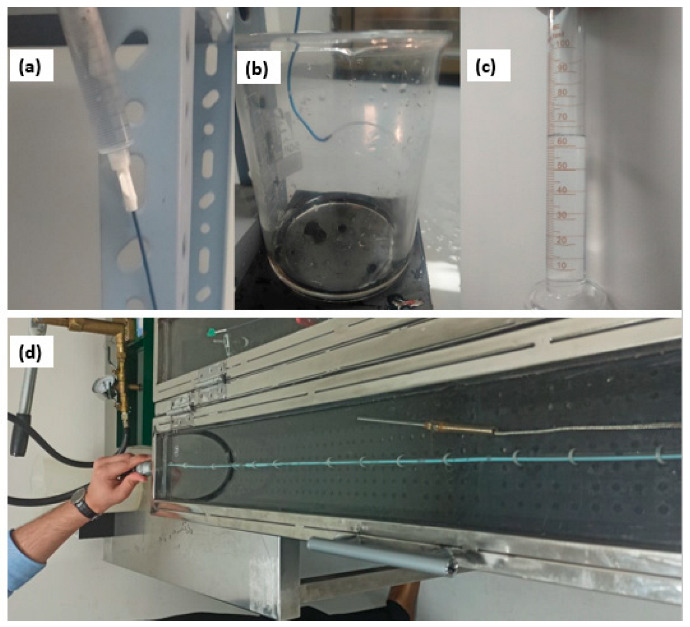
(**a**–**c**) Distilled water is being collected in a 1000 mL borosilicate glass beaker. The collected amount of distilled water was measured volumetrically. (**d**) Catheter is immersed in distilled water maintained at 37 °C.

This test was conducted to ascertain the flow rate of catheters where distal water was allowed to flow through the catheter. The head height of the fluid used was 1000 mL, and the amount of flow was measured volumetrically in a calibrated beaker, as shown in Figure 2a–c. The total flow was measured for 60 s using a digital timer. The PEBAX^®^-based catheters along with BBraun’s Angiodyn^®^, Cordis’ Infiniti^®^, and Terumo’s Radifocus Optitorque^®^ were also subjected to the same pressure.

#### 2.3.3. Test for Burst Pressure under Static Conditions

The purpose of this test was to subject the catheters to a fluid at a constant rate until the product leaked or burst while the device pressure was monitored. The test chamber was filled with distilled water, and 37 ± 2 °C was maintained (Figure 2d). The catheter’s hub was connected to a manual liquid pressure tester, and the distal end was blocked by clamping scissors. A pressure of 1200 psi was created and sustained in the catheter for 15 s. The PEBAX^®^-based catheter and the locally purchased BBraun’s Angiodyn^®^, Cordis’ Infiniti^®,^ and Terumo’s Radifocus Optitorque^®^ were tested under the same parameters.

#### 2.3.4. Determination of Peak Tensile Force

**Figure 3:**
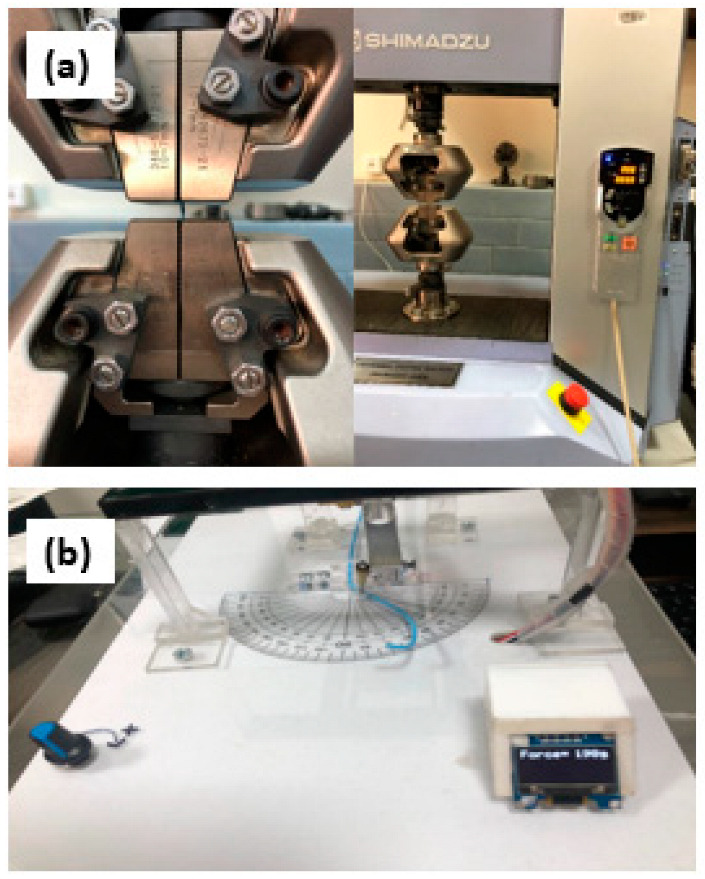
(**a**) Catheter bond strength was tested on UTM, Shimadzu. (**b**) Flexural test bench to test the flexural rigidity of the catheter.

The quality of catheter tube welding is dependent on the use of the appropriate materials, heat supply, and tools’ interface. This test was performed to assess the welding quality and bond strength of welded regions within the developed angiography catheters and to establish the highest tensile force at which these joints and the permanent connections fail. The universal testing machine (UTM) (20 kN) used was from Shimadzu (Kyoto, Japan), which is shown in Figure 3a. It had an appropriate load cell that could examine the force applied to an accuracy of 5% of the claimed value, which is appropriate as per the guidelines given in ISO 10555-1:2013. The diagnostic catheter welded section (25 mm in length) was fixed in the UTM. The force at the failure site was measured after the stress was applied to the joint with a 200 mm/min crosshead speed. The braided–non-braided welded joint was tested to determine its bond strength. The test was carried out in triplicate.

#### 2.3.5. Flexural Rigidity

A catheter tube is passed via arteries with a narrower radius in the coronary vasculature. As the catheter progresses and reaches the coronary arteries, a compressional force is applied by the arteries, resulting in the bending of the catheter and tip, generating stress in the artery lumen. This stress should be kept to a minimum to avoid vessel damage. As a result, the bending moment of the catheter should be relatively low. A catheter with a low flexural stiffness should be advanced into the vasculature to avoid damage to the coronary arteries. A flexural test was performed as per the protocol stated in ISO 25539-2:2020 for the PEBAX^®^-based catheter and commercial samples using the flexural testing bench, as shown in Figure 3b. The length of the catheter taken per sample is 30 mm. The test was performed in triplicates.

#### 2.3.6. Catheter Trackability and Pushability Testing

**Figure 4:**
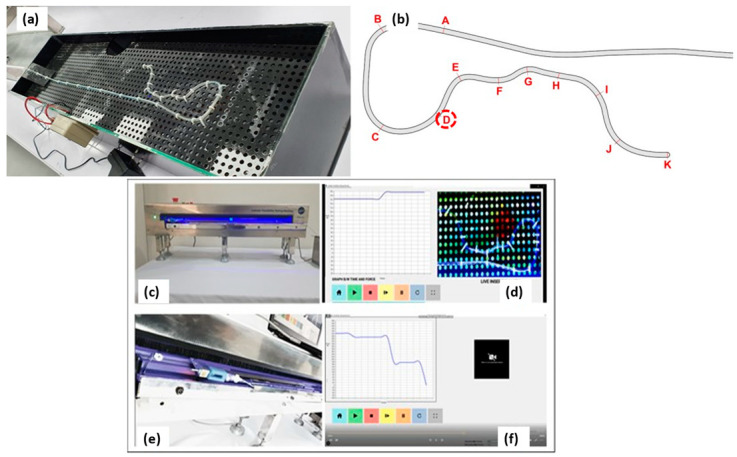
(**a**) Vascular model prepared in the research lab as per ASTM F2394:2017. (**b**) Marked circle represents the end region of the test, where all catheters under test will be stopped and the maximum resistive force experienced by the catheter will be noted. In (**c**,**e**), we exhibit the front and lateral view of the machine; in (**d**,**f**), we exhibit the video and real-time graph generation during test.

A catheter trackability and pushability test was performed to assess the resistive force encountered by the catheter in the vascular model using a catheter trackability test machine (CTTM). The force applied by a clinician to move the catheter to the blockage site is pushability. The catheter shaft is prone to kinking as the push force rises [2]. To evaluate the performance of the PEBAX^®^-, nylon-, and PU-based catheters, a CTTM (Figure 4a–f) was developed inhouse as per ASTM F2394:2017.

CTTM, an in vitro vascular model (mock arterial system, Figure 4), was developed as per the ASTM F2394:2017–07 (2017) Standard Guide for Measuring Securement of Balloon Expandable Vascular Stent Mounted on Delivery System for in vitro characterization. Figure 4a shows a schematic diagram of a vascular model built with a lumen diameter ranging from 1.1 to 4.6 mm [22] in one of NHT’s research labs following the aforementioned standard. A silicone tube with a 2.5 mm inner diameter was employed to replicate the vascular track. The working fluid (buffered saline solution) was prepared to have a pH between 7.35 and 7.45, maintaining slightly alkaline properties to mimic human blood. During the experiment, the temperature was kept at 37 ± 2 °C.

A catheter mounting was attached to a motorized linear guide system to actuate the force applied on the catheter and measure the load/resistance experienced by the catheter when moving in the vascular model via a microcontroller. The catheter was directly attached to the load cell. The guide rail prevented the catheter from bending when put into the vascular model. This equipment assessed the medical device system’s ability to withstand a push force without bending. It evaluated the ease with which a catheter could be driven by measuring the amount of resistance it met, the catheter’s ability to travel along a guidewire during insertion around bending curvatures, and the capacity to transmit torque from one end of the catheter to the other (torqueability). The catheter’s real-time resistance was recorded using a load cell; the data are presented as force–time graphs. The backup directory also recorded and saved the plotted graphs (Figure 4d,f).

The CTTM, developed inhouse to evaluate the performance (resistance experienced) of the PEBAX^®^-based catheters, was used to test coronary angiographic catheters. Track segments were marked with letters A through K. Each segment measured approximately 8 cm obstruction regions of the PEBAX^®^-based catheters, and commercial samples are marked with a red circle in Figure 4b.

The catheters were tested until they reached mark D, as this was the point where the aorta began and the catheter’s distal end entered the heart to flush the media. In usual practice, a coronary artery is only allowed to be subjected to a maximum of 1000 g of force during angiography and angioplasty. The CTTM interface was designed such that it automatically stops if a catheter is subjected to a force of 1000 g.

#### 2.3.7. Statistical Analysis

All experimental approaches were executed in triplicates. Results are represented as mean ± standard deviation, *n* ≥ 3. Statistical analysis was performed to analyze the differences between the experimental results, and a value with *p* < 0.05 was considered significant.

## 3. Results

In this study, a PEBAX^®^-based catheter comprised of a braided shaft was developed for evaluating its performance as a diagnostic angiography catheter. Stainless steel 304, having a diameter of 53 ± 0.01 um, was used for braiding. The inner layer and outer layer of the braided shaft were made of PEBAX^®^ 7233, having a shore hardness of 72D. The non-braided tube at the distal end was made of PEBAX^®^ 5233, having a shore hardness of 55D. The soft tip was made of PEBAX^®^ 3533, which had a shore hardness of 35D. The outer diameter of the whole shaft was 2.00 ± 0.05 mm, and the inner diameter was 1.40 ± 0.05 mm, which equated to 6 French of a diagnostic catheter. The final prototype was obtained after the braided tube was overlap welded to a non-braided tube and a soft tip fused and tapered at the distal end.

### 3.1. Corrosion Resistance Test

**Figure 5:**
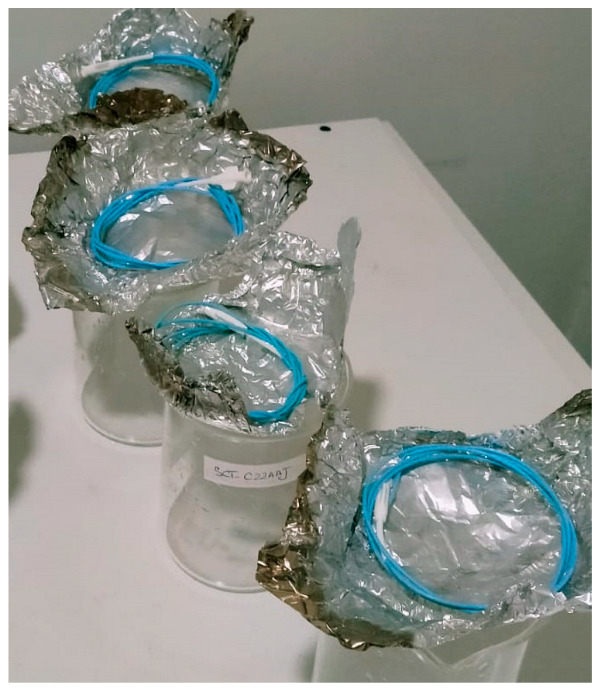
Terumo’s Radifocus Optitorque^®^, Cordis’ Infiniti^®^, BBraun’s Angiodyn^®^, and PEBAX^®^ catheter after corrosion testing; no corrosion was found in any of the abovementioned catheters.

The test was carried out to determine the corrosion resistance of the catheter’s metallic components. The PEBAX^®^-based catheter, together with the Infiniti^®^, Radifocus Optitorque^®^, and Angiodyn^®^, exhibited no signs of corrosion across the entire length of the diagnostic catheter after the experiment was completed. As a result, they were all proven to be corrosion-free, as shown in Figure 5.

### 3.2. Liquid Leakage under Leak Pressure

The tested catheters passed the ‘leak-proof under pressure’ test. The catheter shaft was fixed, the hub was linked to a fixture, and leakage (if any) could be easily visible on the experiment table throughout the leak test. The catheters under testing were extensively evaluated under microscope, and no breaks or leaking were detected.

### 3.3. The Flow Rate through the Catheter

The PEBAX^®^-based catheter had the highest flow rate, followed by Cordis’ Infiniti^®^, BBraun’s Angiodyn^®^, and Terumo’s Radifocus Optitorque^®^. The t-value is 4.24264. The *p*-value is 0.013236. The result is significant at *p* < 0.05. Table 1 shows the flow rate of all the tested catheters.

### 3.4. Test for Burst Pressure under Static Conditions

The catheter hub was linked to a leak-proof connector, and the leak-proof connection was maintained to avoid any leaking. After exposing the catheter to a pressure of 1220 ± 10 psi for 10 s using a manually created liquid pressure tester, no evidence of leaking or thickening across the catheter shaft was observed. During the test, no catheters were broken. Table 2 depicts the burst pressure readings for the tested catheters.

### 3.5. Flexural Rigidity

**Figure 6:**
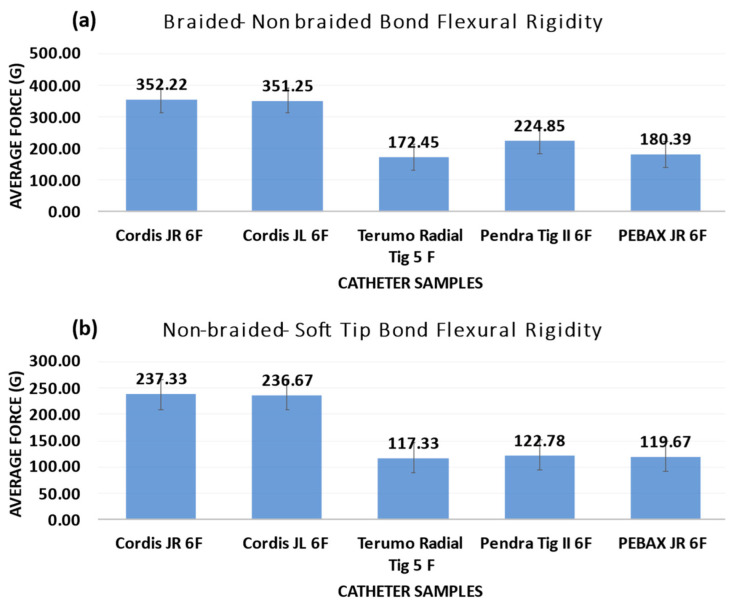
(**a**,**b**) Flexural testing result of PEBAX^®^-based catheter vs. Angiodyn^®^, Infiniti^®^, and Radifocus Optitorque^®^.

The tests for all the catheters were performed in triplicate, and the final value was recorded as an average. BBraun’s Angiodyn^®^ showed the lowest flexural rigidity, followed by the PEBAX^®^-based catheter, Terumo’s Radifocus Optitorque^®^, and Cordis’ Infiniti^®^, as shown in Figure 6a,b, which shows the flexural testing result of the PEBAX^®^-based catheter vs. the Angiodyn^®^, Infiniti^®^, and Radifocus Optitorque^®^.

After completing the test, no kink was found. However, the distal end shapes of the Infiniti^®^ and Radifocus Optitorque^®^ catheters were lost.

### 3.6. Determination of Peak Tensile Force

**Figure 7:**
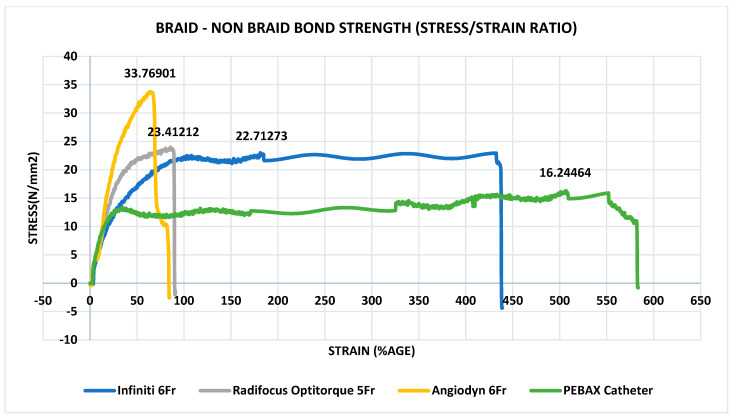
Bond strength of welding bond of braid–non-braid shaft of PEBAX^®^ based catheter, Cordis’ Infiniti^®^, Terumo’s Radifocus Optitorque^®^, BBraun’s Angiodyn^®^. PEBAX^®^ catheter exhibited the maximum strain.

The bond strength of the braided shaft to the non-braided shaft connection was measured in the first test. The braided and non-braided shaft that were overlap weld, made of PEBAX^®^, experienced a force of >16.24 N, and the bond was destroyed at the end of the experiment (Figure 7). According to ISO 10555-1:2013, the bond strength of the catheter’s welded bonds must be greater than 3.0 N. The tensile strength parameters were met by all angiographic catheters, PEBAX^®^-based catheters, Cordis’ Infiniti^®^, Terumo’s Radifocus Optitorque^®^, and BBraun’s Angiodyn^®^.

### 3.7. Quantitative Tracking Analysis: PEBAX^®^-Based Braided Catheter vs. Commercially Available Nylon- and Polyurethane-Based Catheters

**Figure 8:**
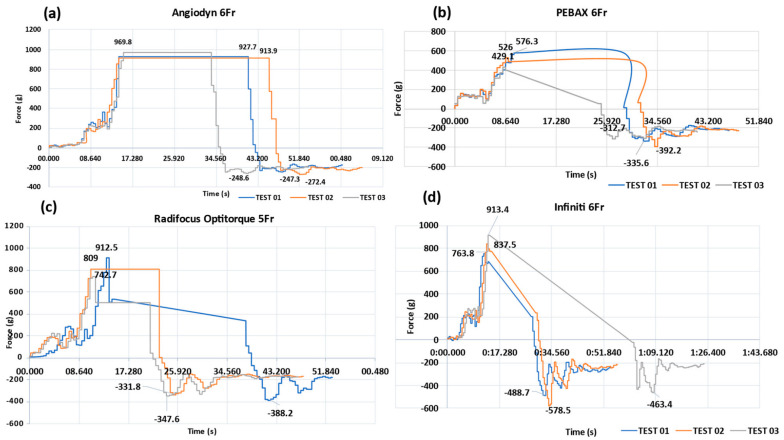
(**a**) Angiodyn^®^ catheter smoothly passed with a max insertion force (average) of 937 g and a max retraction force (average) of −256.1 g. (**b**) PEBAX^®^ based catheter smoothly passed with a max insertion force (average) of 510 g and a max retraction force (average) of −346 g. (**c**) Terumo 5F catheter traversed through the track with a max advancement force (average) of 821.4 and a max retraction force (average) of −355.9 g. (**d**) Infiniti catheter glided through the track with an average insertion and retraction force of 838.1 g and −510.2 g, respectively.

On an automated CTTM, quantitative tracking analysis was performed to determine the force exerted to the catheter as it moved through the designated segments in the mock vasculature. Each catheter was examined three times, and a trend in insertion force (rise or decline) was noted. During catheter insertion, a guidewire was deployed to simulate the human angiography procedure, and the guidewire was withdrawn when the catheter was retracted. The performance of the PEBAX^®^-based catheters was compared with that of the commercially available Terumo’s Radifocus Optitorque^®^, Cordis’ Infiniti^®^, and BBraun’s Angiodyn^®^. Figure 8 depicts the graphs illustrating the quantitative performance of the PEBAX^®^-based braided catheter in comparison with the commercially available nylon- and polyurethane-based catheters.

**Figure 9:**
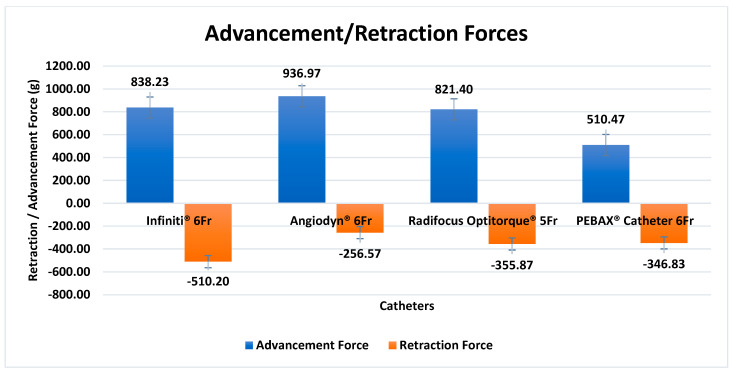
Consolidated trackability results of PEBAX based catheter samples and the commercially available diagnostic angiographic catheter. Data presented in this figure are statistically significant; *p*-value shown in the abovementioned graph is less than 0.05.

When traversing through the mock vasculature, the Angiodyn^®^ (BBraun) catheter smoothly passed through the track until Level C and became stuck there; the maximum advancement force (average) was 937 g, while the maximum retraction force (average) was −256.1 g (Figure 8a). The PEBAX^®^-based catheter smoothly passed through the track until Level D without experiencing resistance and thrust, apparently. The maximum insertion force (average) for this catheter was 510 g, while the maximum retraction force (average) was −346 g (Figure 8b). The Radifocus Optitorque^®^ (Terumo) 5F catheter smoothly passed through the track until Level C and moved forward with thrust until Level D. The maximum advancement force (average) experienced was 821.4 and the maximum retraction force (average) was −355.9 g (Figure 8c). The Infiniti^®^ (Cordis) catheter glided through the track until it reached Level C, then went ahead with push until it reached Level D. The maximum insertion force (average) experienced was 838.1 g, while the maximum retraction force (average) experienced was −510.2 g (Figure 8d). The consolidated results of the advancement and retraction forces are mentioned in Figure 9.

## 4. Discussion

The PEBAX^®^-based diagnostic angiographic catheter was developed using extrusion methods, where PEBAX^®^ was extruded on a round-wire braid and was also used as an inner liner and where the distal tube shaping was performed on a thermoforming machine using an aluminum die. An atraumatic tip was developed using PEBAX^®^-3533, having a 35D shore hardness. The PEBAX^®^-based catheter results provide evidence that the catheter showed better performance during in vitro testing along with optimal flexural rigidity and minimal advancement force and that it has the potential for use as a prominent angiographic device for diagnostic purposes. There was no study in the literature that compared and/or reported the performance of PEBAX-, nylon-, and polyurethane-based catheters. Therefore, this study was intended to evaluate the performance of a PEBAX^®^-based angiographic catheter by comparing it with three commercially available diagnostic angiographic catheters. This research concentrated on the in vitro testing and outcomes of PEBAX^®^-based diagnostic angiographic catheters rather than the novelty of PEBAX^®^ as a material for catheter assembly [23].

PEBAX^®^-based catheters are extensively used in urethral catheters such as urodynamics catheters [24]; however, PEBAX^®^ has not been excessively used in the construction of coronary catheters. The purpose of this study was to see if PEBAX^®^-based coronary catheters are comparable to currently available nylon- and polyurethane-based catheters. The PEBAX^®^-based diagnostic angiographic catheter is built and optimized to meet the angiography needs of coronary artery patients in the native population. The angiography catheter traversing through the femoral and radial routes to the coronary arteries can deliver radiopaque media. The PEBAX^®^-based catheter was engineered, in this research, to be more flexible to glide in and out of the artery without causing discomfort. PEBAX^®^ is a robust thermoplastic polymer with low moisture absorption properties, making polymers less prone to cracks. It is a biocompatible polyester–amide copolymer, having little to no cytotoxicity with outstanding mechanical properties that ensure its usability in manufacturing catheters with very thin walls. A catheter with a reduced profile causes less stress to the coronary vessels [25,26,27].

In this study, PEBAX^®^-, nylon-, and polyurethane-based catheters were put through six different types of tests per the ISO 10555-1:2013/Amd 1:2017 standard [18]. The PEBAX-based catheter exhibited favorable results, making it suitable for its intended usage. The burst pressure of a PEBAX^®^-based 6 French catheter in a static situation is 1220 psi, similar to that of Infiniti^®^ (Cordis), Angiodyn^®^ (BBraun), and Radifocus Optitorque^®^ (Terumo). Diagnostic angiographic catheters typically have a burst pressure endurance of 1000 psi to 1200 psi [25]. When the dye is pumped into the coronary arteries at a greater pressure, the catheter’s burst pressure endurance property ensures the safety of the catheter and the patient. The PEBAX^®^-based catheter meets the standards and withstands high pressures, making it appropriate for power injections [28,29].

When a diagnostic angiographic catheter is inserted into the aorta to deliver media to the coronary artery, the catheter tubing must be turned around until the catheter tip is positioned in the desired vessel. This rotation must be regulated such that the catheter’s proximal end transfers stresses to the distal end, allowing the catheter’s tip to be positioned in the appropriate conduit. Before a diagnostic catheter is deflected, it lies vertically against the vessel wall and exerts its force. The catheter tube may buckle due to the compressional stress exerted by the vascular walls on the catheter. To reduce the catheter-induced trauma, minimal buckling force exhibition to the vessels is anticipated [30]. When the braided–non-braided welding point of a PEBAX^®^-based 6 French catheter was tested, it showed a flexural rigidity of 135 g. In contrast, Cordis’ Infiniti 6 French catheter offers a maximum flexural rigidity of 188 g. The flexural rigidity of the BBraun’s Angiodyn^®^ was 95.0 g, and Terumo’s Radifocus Optitorque^®^ demonstrated a 145.0 g force. The catheter will be more flexible if the flexural rigidity value is lower. The flexural rigidity of a PEBAX^®^-based catheter is comparable to that of commercial catheters. When the flexural stiffness of non-braided shaft, soft tip catheters were tested, BBraun’s Angiodyn^®^ was revealed to be the most flexible of all [30]. The PEBAX^®^-based catheter flexural rigidity was almost comparable to Terumo’s Radifocus Optitorque^®^. When exposed to stress and moved to a 45 degree angle, Infiniti^®^ demonstrated maximum flexural rigidity. The flexural rigidity of the catheter rises as the wall thickness of the catheter increases. Thinner walls on diagnostic angiographic catheters, on the other hand, are indicated to reduce flexural rigidity. The thin walls of the PEBAX^®^-based catheters had superior flexural rigidity results compared to Terumo’s Radifocus Optitorque^®^ and Cordis’ Infiniti^®^, making the PEBAX^®^-based catheters a viable angiographic catheter competitor [14,17,31].

At the braided–non-braided shaft junction, a PEBAX^®^-based catheter had a bond strength of >16.20 N. The acceptable standard value for catheter bond strength is >3.0 N. As a result, the PEBAX^®^ catheter’s bond strength was almost five times stronger than the established threshold [32]. Hence, the PEBAX^®^ catheter is unlikely to break or damage when subjected to compressional arterial forces. Figure 7 shows the tensile stress–strain curve of PEBAX^®^-based and commercial catheters using the ISO 10555-1: 2013 specifications. Cordis’ Infiniti^®^, 6 French, has a bond strength of 22.71 N compared to other commercial catheters. Terumo’s Radifocus Optitorque^®^ has a bond strength of 23.4 N before breaking. BBraun’s Angiodyn^®^ exhibited a bond strength of 33.76 N before breaking. As a result, the tensile strength values of PEBAX^®^-based catheters are better than the acceptable range and are depicted in the stress–strain graph [32].

Flow rates were measured in triplicates, as suggested by ISO-10555-1:2013. The 6 French catheters were used in the experiment. The catheter’s wall thickness is critical in determining whether the catheter’s flow rate increases or decreases. The catheter’s flow rate is inversely related to its wall thickness. The lower the wall thickness, the greater the flow velocity while maintaining a fixed outside diameter. PEBAX^®^-based catheters had the highest flow rate in this study, followed by Cordis’ Infiniti^®^, BBraun’s Angiodyn^®^, and Terumo’s Radifocus Optitorque^®^. Because of its improved ability to extrude through narrow walls, PEBAX^®^ is a desirable material for angiographic catheters [15,17].

The PEBAX^®^ series of catheter jacketing materials has an advantage because its polyether–block–amide composition allows quick customization. The PEBAX^®^ family is beneficial in catheter building applications because it combines desirable qualities such as pushability, flexibility, and tailorability [14]. PEBAX^®^ performs exceptionally well in cytotoxicity tests due to its inert behavior. As a result, it is ideal for critical dye transfer applications where any disturbance of blood cells might result in negative consequences [25].

PEBAX^®^ is commonly used as a urethral catheter material because of its chemical inertness and ability to generate multi-durometer lengths. It also has a slightly greater flexural modulus than other TPEs; when used in high durometers, it performs better in kink resistance tests. PEBAX^®^ may be reheated and used to create various components for a continuous extrusion along the catheter length. Since a catheter’s hardness may vary throughout its length, tiny parts of hard PEBAX^®^ can be used before switching to a soft PEBAX^®^ grade [15,30].

Compared to Nylon 12 and polyurethane, the following two attributes of PEBAX^®^ were studied: flexural modulus and tensile strength. Flexural modulus measures a material’s stiffness that may be compared. According to mathematical models, using shore hardness as a replacement for the flexural rigidity of a material is acceptable. The material will be more rigid if the shore hardness is greater. The stiffness and performance of the catheters are determined by the polymeric material’s intrinsic rigidity and the cross-sectional catheter design [33,34,35].

**Figure 10:**
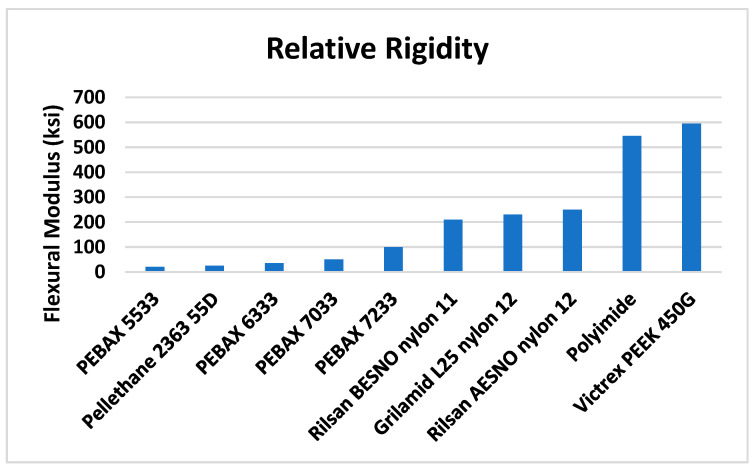
Flexural modulus of various polymers considered for catheter construction [36].

Sarwar et al. indicated that PEBAX^®^ had a lower flexural modulus than other medical grade polymers, such as Nylon 11,12 and polyimide, as shown in Figure 10 [26,34]. PEBAX^®^ can be preferable for coronary angiographic catheters due to its lower flexural stiffness [35]. The less stiff the catheter is, the simpler it will be to glide through the artery. The PEBAX^®^ catheter exhibited an advancement force of 510 g. Angiodyn^®^, Infiniti^®^, and Radifocus Optitorque^®^, on the other hand, had maximum advancement forces of 936.9 g, 838.2 g, and 821.4 g, respectively.

The direct relationship between the catheter’s burst pressure and tensile tension must be seen. As a result, the tensile strength of polymeric materials can be used to determine the burst pressure for a catheter tube with a particular cross-sectional area. PEBAX^®^ exhibits a tensile strength comparable to nylon, depending on the durometer employed in the catheter [35]. The proximal shaft of the catheter is required to endure 1200 psi pressure. The proximal end of the catheter is made of PEBAX^®^ with a 72D shore hardness to provide injection pressure endurance [14,31].

In vivo vascular anatomy provides the desired environment for validating the use of a device for an interventional procedure [36]. Nevertheless, intrinsic risk, complicated preparations, and increasing costs restrict its use [37]. In vitro vascular models have proven to be a popular platform for such procedures, which is why the mock vascular model was created with silicone tubes that mimic the inner diameter of a human cardiovascular artery. The vessel’s coefficient of friction values is recreated in in vitro device testing equipment, which is needed to explore the surface integrity further and obtain safety data for any long-term biological consequences [38]. Trackability is the force required by the catheter to reach the lesion to be treated. It is assumed that the trackability is influenced by the pushability of a delivery system [38]. The catheter trackability testing machine (CTTM) was built by the N-ovative Health Technologies (NHT) research team to evaluate the angiographic catheter’s performance characteristics. The pushability of the PEBAX^®^-based braided angiographic catheters was examined along curved channels for further assessment. The results were compared to those of commercially available angiographic catheters in Pakistan, namely BBraun’s Angiodyn^®^, Cordis’ Infiniti^®^, and Terumo’s Radifocus Optitorque^®^. PEBAX^®^ braided catheters had a significant force difference from commercially available nylon- and PU-based catheters. The PEBAX^®^-based braided catheter experienced less advancement force, which was 510 g (average of three tests). As the shear stress reaches a certain value, it can damage the vascular wall. There is a limited amount of information about trackability and pushability forces in the literature. All commercial diagnostic angiographic catheters, including the Infiniti^®^, Radifocus Optitorque^®^, and Angiodyn^®^, experienced maximal force in the C–D area. When compared to other commercial samples, the PEBAX^®^-based catheter yielded promising results. With 510 g, it experienced the maximum peak force at the D bend, while commercial samples had peak forces before reaching the D bend. At the C bend, BBraun’s Angiodyn^®^ encountered a maximum force of 936.9 g, followed by 838.2 g and 821.4 g for Cordis’ Infiniti^®^ and Terumo’s Radifocus Optitorque^®^, respectively. The greatest retraction force was 511.9 g for Cordis’ Infiniti^®^, while BBraun’s Angiodyn^®^ exhibited the least retraction force.

To summarize the findings, coronary arteries can tolerate forces of approximately 1000 g (10 N) [39], and PEBAX^®^-based braided catheters had significantly less advancement forces when traversing the coronary arteries than nylon- and PU-based catheters. This extensive testing demonstrated that a PEBAX^®^-based catheter can inject more radiopaque material to coronary arteries while causing minimal arterial spasm. Interventional cardiologists strive for a flexible catheter with minimal advancement forces and better flexural rigidity and bond strength. PEBAX^®^-based catheters are a promising choice for such circumstances. Analyzing the effects of wall friction and tortuosity during the advancement of catheters in vascular arteries will provide more insight into the performance of catheters.

## 5. Conclusions

In this research, a PEBAX-based diagnostic catheter was developed and evaluated in vitro to compare its performance with the commercially available nylon- and polyurethane-based catheters for angiography. The catheters were tested for corrosion resistance, being leak-proof, burst pressure endurance, flexural rigidity, bond strength, and quantitative trackability using an automated catheter trackability testing machine (CTTM). The PEBAX-based catheter was corrosion-free, exhibited enhanced bond strength, and endured 1220 psi pressure. The PEBAX-based catheter exhibited the least bond-flexural rigidity (180.4 g) among the 6 French (nylon- and PU-based) catheters. It also exhibited the least advancement force (510.4 g) in comparison with the nylon- and polyurethane-based catheters when traversing through the mock arterial system. This extensive testing demonstrated that a PEBAX^®^-based catheter can inject more radiopaque material (because of the enhanced flow rate) to the coronary arteries while causing minimal arterial spasm, owing to the minimum advancement force it requires. This makes this catheter a promising option for avoiding vascular spasms during diagnostic procedures, imperative before a percutaneous coronary intervention. Clinical trials need to be conducted to better ascertain the potential of PEBAX^®^-based catheters as tools in angiography procedures.

## Figures and Tables

**Table 1:** Flow rates of 6 French catheters.

Brand	Catheter	Type	Length of the Catheter (cm)	Gravity Flow Rate (mL/min)
Cordis	Infiniti^®^	Judkins Left	100.4	62.0
Cordis	Infiniti^®^	Judkins Right	101.1	60.2
BBraun	Angiodyn^®^	Tig II	103.2	60.2
Terumo	Radifocus Optitorque^®^	Radial Tig	100.2	60.2
PEBAX	PEBAX^®^-based braided catheter	Judkins Left and Judkins Right	100.2	63.0

**Table 2:** Burst pressure values for catheters.

Details of Catheters Tested for Burst Pressure					
Brand	Catheter	French Size	Time (s)	Pressure Generated	Results
Cordis	Infiniti^®^	6	10	1210	No burst or bulging was observed
Cordis	Infiniti^®^	6	10	1210	No burst or bulging was observed
BBraun	Angiodyn^®^	6	11	1200	No burst or bulging was observed
Terumo	Radifocus Optitorque^®^	6	11	1200	No burst or bulging was observed
PEBAX	PEBAX^®^-based Catheter	6	12	1220	No burst or bulging was observed

## Data Availability

Data will be made available upon request.
